# A phase II study of cisplatin with intravenous and oral vinorelbine as induction chemotherapy followed by concomitant chemoradiotherapy with oral vinorelbine and cisplatin for locally advanced non-small cell lung cancer

**DOI:** 10.1186/1471-2407-14-231

**Published:** 2014-03-30

**Authors:** Delphine Lerouge, Alain Rivière, Eric Dansin, Christos Chouaid, Cécile Dujon, Roland Schott, Armelle Lavole, Vincent Le Pennec, Elizabeth Fabre, Jacky Crequit, Francis Martin, Stéphanie Dehette, Pierre Fournel, Bénédicte Precheur-Agulhon, Eric Lartigau, Gérard Zalcman

**Affiliations:** 1 Department of Oncology radiotherapy, CRLCC F. Baclesse, Caen, France; 2 Department of Pneumology, Centre O. Lambret, Lille, France; 3 Department of Pneumology, Centre hospitalier Intercommunal de Créteil, Créteil, France; 4 Department of Oncology, Hôpital A. Mignot, Le Chesnay, France; 5 Department of Pneumology, Centre P. Strauss, Strasbourg, France; 6 Department of Pneumology, Hôpital Tenon, Paris, France; 7 Department of Radiology, Centre Hospitalier Universitaire de Caen, Caen, France; 8 Department of Medical Oncology, Hôpital européen G Pompidou, Paris, France; 9 Department of Pneumology, Centre Hospitalier de Creil, Creil, France; 10 Department of Pneumology, Centre hospitalier, Compiègne, France; 11 Department of Pneumology, Institut de Cancérologie Lucien Neuwirth, Saint-Etienne, France; 12 Institut de Recherche Pierre Fabre, Boulogne-Billancourt, France; 13 Deparment of Pneumology and Thoracic Oncology, Centre Hospitalier Universitaire de Caen, France

**Keywords:** Locally advanced non-small cell lung cancer, Oral vinorelbine, Chemoradiotherapy

## Abstract

**Background:**

Concomitant platinum-based chemotherapy and radiotherapy (CT-RT) is the recommended treatment for unresectable locally advanced stage III non-small cell lung cancer (NSCLC). We conducted a phase II study to evaluate the efficacy and safety of fractionated oral vinorelbine with cisplatin as induction CT followed by CT-RT.

**Methods:**

Patients with stage III NSCLC received 2 induction cycles of intravenous vinorelbine 25 mg/m^2^ and cisplatin 80 mg/m^2^ on day 1 and oral vinorelbine 60 mg/m^2^ on day 8. Responding patients received 2 more cycles of cisplatin 80 mg/m^2^ on day 1 and oral vinorelbine 20 mg on days 1, 3 and 5 concomitantly with radiotherapy 2 Gy daily, 5 days/week for a total of 66 Gy.

**Results:**

Seventy patients, median age 61 years, were enrolled. Overall response rate (ORR) was 50.0%; Disease Control Rate was 81.42%. Median PFS was 14.58 months [95% CI, 10.97-18.75]. Median OS was 17.08 months [95% CI, 13.57-29.57]. One-year and 2-year survival rates were 68.6% [95% CI, 57.7-79.4] and 37%. One patient had a grade 3 pulmonary radiation injury and 26.5% had graded 1/2 esophagitis.

**Conclusion:**

In non-operable IIIA-IIIB NSCLC, the combination oral vinorelbine (fractionated fixed dose) plus cisplatin, during concomitant CT-RT, could offer a well-tolerated option, with comparable activity to I.V. vinorelbine-based chemoradiotherapy regimens.

**Trial registration:**

ClinicalTrials.gov, NCT01839032

## Background

Lung cancer is a global public health issue worldwide, with 1.38 million of deaths in 2008 [1]. Non-small cell lung cancer (NSCLC) is the predominant histological type accounting for nearly 85% of all lung cancers [2].

At diagnosis, at least, one third of patients have a locally advanced (stage III) disease [3].

Most patients with stage IIIA (N2) and IIIB NSCLC cannot undergo complete surgical resection. Up-front concomitant platinum based chemotherapy and radiotherapy are currently recommended [4,5]. Cisplatin-based chemotherapy increases radiotherapy-induced cell lethal DNA lesions, along with eradication of distant micrometastases and some other cytotoxic e.g. vinorelbine have a supra-additive effect by inducing cell cycle synchronization into the radiosensitive G2M phase.

Vinorelbine is a semi-synthetic vinca alkaloid approved for the treatment of NSCLC and breast cancer [6] available in intravenous and oral formulations, oral vinorelbine provides similar efficacy to intravenous vinorelbine, while offering benefits in terms of convenience, ease of administration, as well as satisfying patient preference [7,8].

The combination vinorelbine/cisplatin is considered as one of the standard regimens for concomitant CT-RT in unresectable locally advanced stage III NSCLC [4]. An optimal efficacy/tolerance ratio was achieved with vinorelbine/cisplatin when compared with gemcitabine/cisplatin or paclitaxel/cisplatin [9]. We aimed to evaluate, the tolerance and efficacy, a new schedule of oral fractionated vinorelbine formulation, at fixed dosing with cisplatin, concomitantly with radiotherapy, following induction chemotherapy with vinorelbine/cisplatin in unresectable stage III NSCLC.

## Methods

This phase II, multicenter, single-arm open-label study was conducted in France. Patients with histologically or cytologically confirmed stage IIIA (only N2), and dry IIIB previously untreated inoperable NSCLC, 18 to 75 years old, Karnofsky Performance Status (KPS) ≥ 80%, weight loss ≤ 10% within the previous 3 months, and normal organ functions were eligible. They had at least one measurable lesion according to Response Evaluation Criteria in Solid Tumors (RECIST version 1.0) [10].

The study was approved by the ethics committee (CPP Nord-Ouest IV) and was registered in clinicaltrials.gov (NCT00295672). It was conducted in accordance with the declaration of Helsinki and in compliance with Good Clinical Practice guidelines. All patients provided informed consent prior to any study procedure.

### Treatment plan

#### Chemotherapy

During the induction period (IP), patients received chemotherapy for two 3-week cycles. Bolus intravenous vinorelbine 25 mg/m^2^, was administered on day 1, then cisplatin 80 mg/m^2^ was administered over 1-hour infusion. Oral Vinorelbine 60 mg/m^2^ was also administered on day 8.

**Figure 1:**
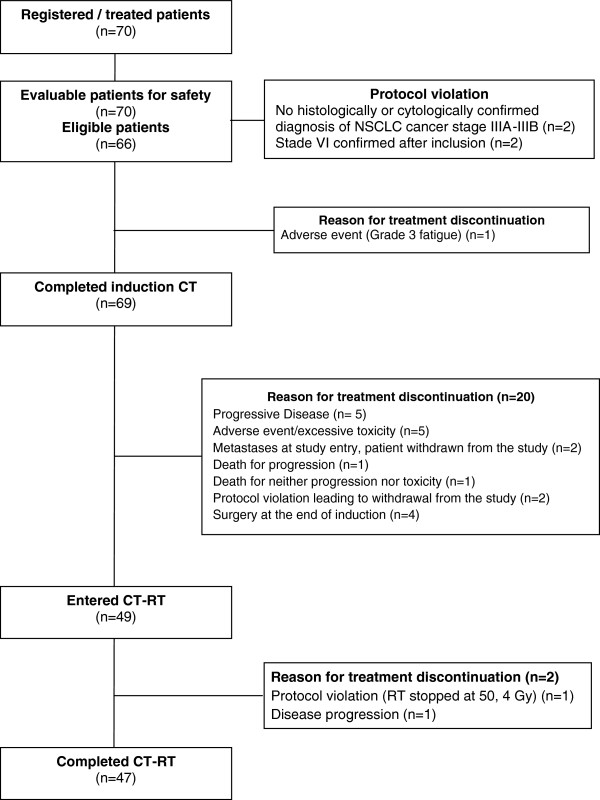
Patients disposition.

**Figure 2:**
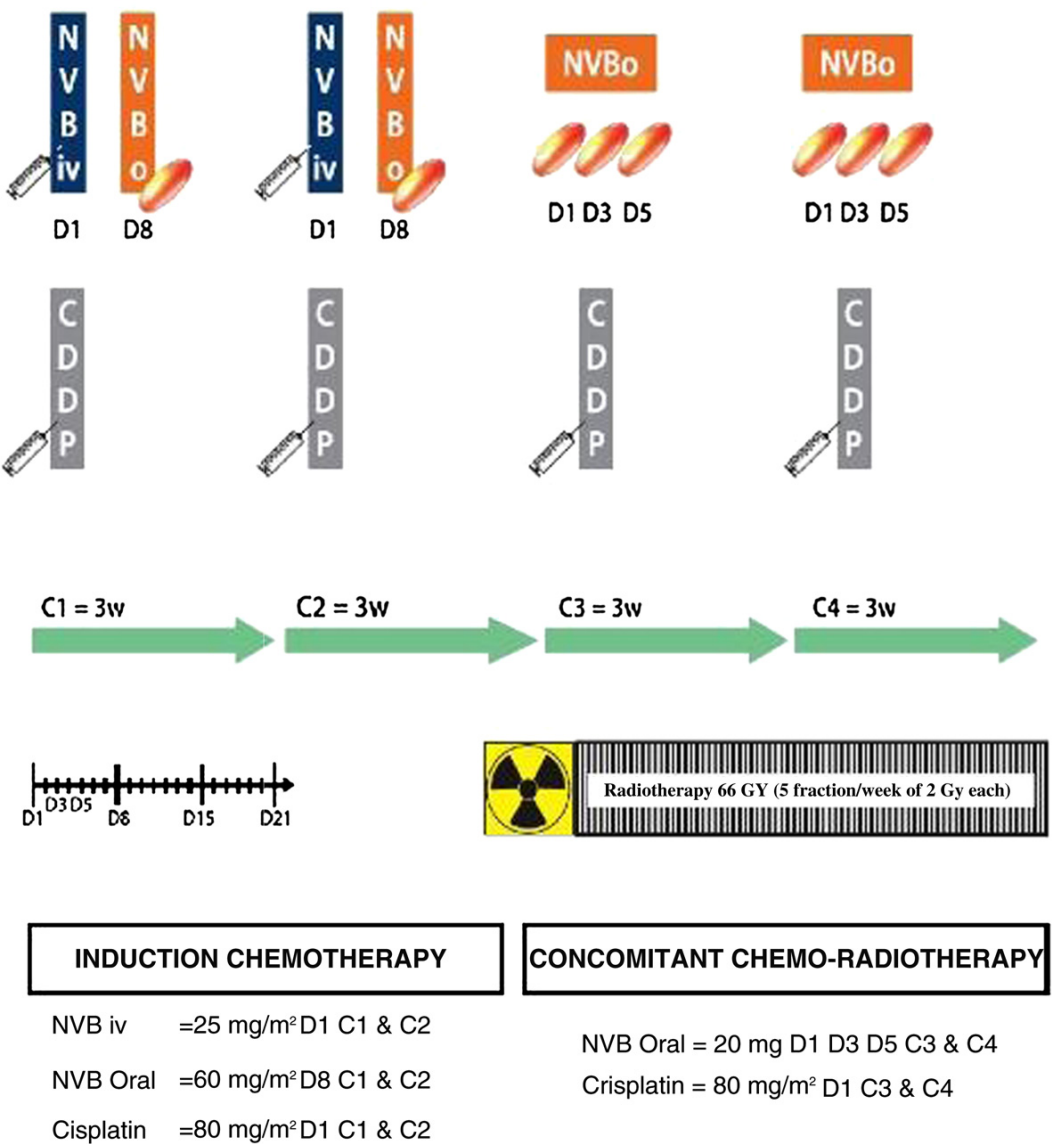
Treatment schedule.

Patients with progressive disease (PD) at the end of the IP were withdrawn from the study, but followed for survival assessment. Patients with objective response (OR) or no change (NC) continued the concomitant period (CP) including two additional 3-week cycles of chemotherapy with concomitant radiotherapy (Figure 1). During CP, vinorelbine was administered at the fractionated oral dose of 20 mg on days 1, 3, and 5. Cisplatin 80 mg/m^2^ was administered on day 1 (Figure 2).

The administration of vinorelbine was delayed by one week for an absolute neutrophil count (ANC) <1.5 × 10^9^/L and/or platelet count <75 × 10^9^/L. Treatment with growth factors was allowed for febrile neutropenia or neutropenic infection. Vinorelbine was postponed or stopped for neurological toxicity graded ≥ 2 or liver toxicity. The cisplatin dose was reduced by 50%, postponed or stopped based on creatinine clearance. Symptomatic ototoxicity led to 50% reduction of cisplatin dose.

#### Radiotherapy

Radiotherapy, 2 Gy fractions daily, 5 days a week for a total dose of 66 Gy started on day 1 of the CP. 3D-RT was delivered with a curative intent with a 3D conformal field. Target volumes were delineated according to the International Commission on Radiation Units and Measurements-62 (ICRU 62): Gross Tumor Volume (GTV) included the tumor (GTV t) and involved nodes (GTV n). The Clinical Target tumor Volume (CTV t), was automatically generated by adding a 5 to 8 mm 3D expansion to the GTV. The Clinical Target tumor and node Volume (CTV t + n) was the addition of CTV t, ipsilateral hilum and ipsilateral mediastinum. The ipsilateral supra clavicular fossa was included only if the tumor was located in the upper lobe. The Planning Target Volume (PTV) was obtained by adding a minimum of 10 mm 3D expansion to CTV. The structures of interest were delineated on dosimetric CT scans. The target goal for the PTV t + n was that ≥95% of PTV t + n receives 100% of the prescribed dose, but no more than 107%.

Radiotherapy was discontinued in case of patient’s decision, progression, side effects or comorbidities non-compatible with radiation, ANC <500/mm^3^ and/or febrile neutropenia and/or platelets <50,000 mm^3^.

### Study assessments

Tumor assessments were performed at the end of the IP and at the end of the CP. Patients were followed for safety during the treatment and 30 days after the last treatment administration: Laboratory tests were performed before each cycle. Toxicity was graded according to the National Cancer Institute Common Toxicity Criteria (NCI CTC) version 2.0. A complete clinical examination was performed at the end of each cycle. Tumor response was assessed according to RECIST criteria version 1.0, every 3 months for 2 years and then every 6 months until death. Unconfirmed responses were considered as NC.

CT-scans of responders and borderline patients were reviewed by an expert thoracic radiologist.

### Endpoints and statistical analysis

Fleming one-sample multiple-testing procedure for phase II clinical trials was used [11]. A total of 65 patients were enrolled to obtain 60 evaluable patients allowing for a type I error of less than 5% and type II error of less than 10% with a null hypothesis (H0) of 50% and an alternative hypothesis (H1) of 70%.

The primary endpoint was best OR according to RECIST criteria in evaluable patients. The OR rate (ORR) was expressed as the sum of the percentage of complete response (CR) and partial response (PR). The Disease Control Rate (DCR) was defined as the sum of the OR and the NC rates.

Secondary endpoints were progression-free survival (PFS), and overall survival (OS), calculated by the Kaplan-Meier method in the Intent to Treat Population (ITT), and duration of response (DR). The safety analysis reported the worst grade of the adverse events (NCI CTC v. 2.0) for the safety population.

## Results

### Patients

A total of 70 patients were enrolled between October 2005 and May 2008 and 69 completed induction therapy. Among the 49 patients who started consolidation treatment, 47 patients completed concomitant CT-RT. The reasons for treatment interruption were protocol deviation (n = 1) and tumor progression (n = 1) (Figure 1).

All included patients were evaluable for safety and 64 for best tumor response.

Overall, 84.3% were men, median age was 61 years [39.5-73.8] and median KPS was 90% [80-100%]. Approximately, 2/3 of patients had stage IIIB disease (Table 1).

**Table 1:** Patient characteristics at baseline (ITT population, n = 70)

	**n (%)**
**Median age, years (range)**	**61 (39.5-73.8)**
35–49 years	10 (14.3%)
50–64 years	36 (51.4%)
≥ 65 years	24 (34.3%)
**Gender**	
Men	59 (84.3%)
Women	11 (15.7%)
**Median performance status (KPS)**	90
80%	25 (35.7%)
90%	12 (17.1%)
100%	33 (47.1%)
**Histology**	
Squamous cell	31 (44.3%)
Adenocarcinoma	23 (32.9%)
Large cell Carcinoma	13 (18.6%)
Giant cell carcinoma	3 (4.3%)
**Staging**	
IIIA	20 (28.6%)
IIIB	48 (68.6%)
IV*	2 (2.9%)
**Median delay between diagnosis and study entry, months (range)**	1 (0.2-6.4)

*Stage IV: Dubious at inclusion, was confirmed at following evaluations.

### Drug exposure

A total of 237 cycles were administered during a median duration of treatment of 12.4 [3-15] weeks. Forty nine patients (70%) received the planned 4 cycles (see Table, Additional file 1, which details drug exposure).

During the IP, the median dose intensity (DI) and median relative DI were 18.2 mg/m^2^/week and 90.8% for oral vinorelbine respectively and 7.8 mg/m^2^/week and 94.2% for intravenous vinorelbine. During the CP, they were 20 mg/m^2^/week and 100% for vinorelbine. Thirty five patients (50.7%) had at least one cycle delayed by ≥4 days. Such delays were recorded for 43 (25.7%) cycles; only 13 were due to drug related toxicities (n = 12).

The median dose of radiotherapy was 66 Gy [50–67].

### Efficacy

The Best Overall Response was assessed by an independent thoracic radiologist: 5 patients achieved a CR (7.14%), 27 patients were in PR (38.57%) and 25 had NC (35.5%), leading to an ORR of 50.0% [95% CI, 37.2-58.1], leading to reject the H0 null hypothesis of inefficacy, according to the statistical design. Disease Control Rate (DCR) was 81.43% [95% CI, 70.34-89.72] (Table 2). Four patients became operable at the end of the IP. Two patients died at 7 months and 16 months after surgery, 2 patients were still alive (16 and 33 months after surgery) at the time of the cut-off.

**Table 2:** Best Overall Response (Evaluable population, n = 64)

	**Evaluable population n = 64**
Complete response	5 (7.81%)
Partial response	27 (42.18%)
No change	25 (39.06%)
Progression disease	8 (12.5%)
Non evaluable*	6 (9.37%)
Objective response rate	32 (50.0%)
Disease control rate	57 (89.06%)
Median duration of the response**	Not reached

All scans of responding and/or border line stable patients were reviewed by an independent panel review.

*5 NE: (2 patients were not eligible, 3 patients had adverse events avoiding evaluation, for 1 patient, RECIST measurements were never obtained since initial CT-scan was lost).

**Median Duration of the response was not reached at the cut-off date (29/12/2009).

At the analysis performed on September 29th, 2009, the median DR was not reached meaning that more than 50% of patients were still controlled at a median follow-up of 19.08 months [95% CI, 16.00-24.11].

Disease progression was reported for 51 patients. Relapses were loco-regional for 16 patients (22.9%), distant for 21 (30%) and, mixed for 5 (7.1%). The most frequent sites of disease progression were brain (17.1%), liver (8.6%) and bone (8.6%) (see Table, Additional file 2, which detailed sites of disease progression). Nine patients (12.9%) had a clinical progression, including general health deterioration (n = 2), respiratory insufficiency (n = 2), cataclysmic hemoptysis (n = 1), cataclysmic hemorrhage (n = 1), general fatigue (n = 1). No more details were reported for 2 patients.

**Figure 3:**
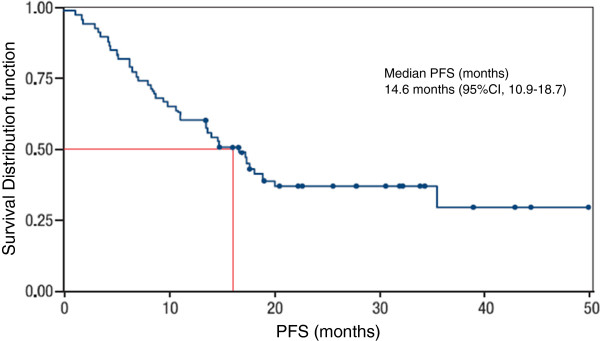
Progression free survival (ITT).

Median PFS was 14.58 months [95% CI, 10.97-18.75] (Figure 3) and PFS rate at 12 months was 58.6% [95% CI, 47–70.1] (Table 3).

**Table 3:** PFS and survival results (ITT population)

	**ITT population n = 70**
Median progression free survival, months	14.58 [95% CI; 10.97-18.75]
Progression free survival rate at 12 months, %	58.6 [95% CI; 47.0-70.1]
Median overall survival, months	17.08 [95% CI; 13.56-29.57]
Survival rate at 6 months, %	88.6 [95% CI; 81.1-96.0]
Survival rate at 12 months, %	68.6 [95% CI; 57.7-79.4]
Survival rate at 18 months, %	43.4 [95% CI; 31.3-55.6]
Survival rate at 24 months, %	>37*

*95% CI was not estimated (data were not mature).

**Figure 4:**
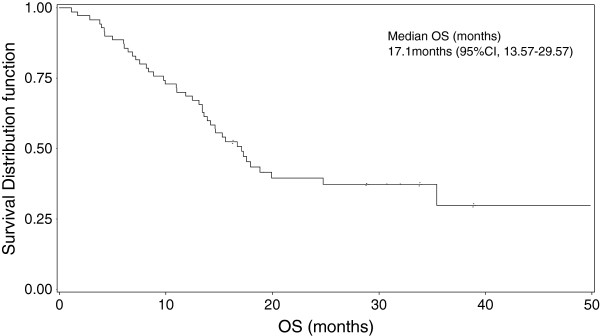
Overall survival (ITT).

At a median follow-up of 37 months, median OS was 17.08 months [95% CI, 13.57-29.57] with 1-year survival rate of 68.6% [95% CI, 57.7-79.4] (Figure 4). The 2-year survival rate was 37% (Table 3). Survival rates were higher in squamous (*vs*. non-squamous cell cancer patients) and stage IIIA patients (*vs*. IIIB patients) (see Table, Additional file 3, Overall survival rates according to histological type and disease stage).

### Safety

During the IP, Grade 3–4 leucopenia and neutropenia were reported for 8.6% and 24.3% of patients respectively. Their incidence decreased to 2% each during the CP. Esophagitis was reported in 13 patients (26.5%), all grade 1–2. One patient had a grade 3 pulmonary radiation injury, he died 4 months after the start of the event and one patient had a grade 1 radiation pneumonitis. Safety results are detailed in Table 4.

**Table 4:** Hematological, clinical and radiation toxicities per patient (NCI/CTC) (ITT population, n = 70)

	**Induction therapy (n =70), n (%)**		**Consolidation therapy (n = 49), n (%)**	
	**All grades**	**Grade 3-4**	**All grades**	**Grade 3-4**
**Hematological toxicities per patient**				
Anemia	36 (51.4)	-	39 (79.6)	2 (4.1)
Neutropenia	31 (44.3)	17 (24.3)	20 (40.8)	1 (2)
Leukopenia	27 (38.6)	6 (8.6)	30 (61.2)	1 (2)
Thrombocytopenia	15 (21.4)	-	11 (22.4)	-
Febrile neutropenia (Pizzo)	2 (2.85)		-	
**Clinical toxicities (NCI-CTC)**				
Fatigue	31 (44.3)	2 (2.9)	23 (46.9)	-
Nausea	29 (41.4)	1 (1.4)	15 (30.6)	1 (2)
Vomiting	18 (25.7)	2 (2.9)	10 (20.4)	1 (2)
Diarrhea	11 (15.7)	-	5 (10.2)	-
Constipation	9 (12.9)	-	2 (4.1)	-
Anorexia	6 (8.6)	-	3 (6.1)	1 (2)
Hypercreatininemia	1 (1.4)	-	-	-
**Radiation toxicities**				
Esophagitis	-	-	13 (26.5)	-
Skin injury	-	-	12 (24.5)	-
Dysphagia	-	-	6 (12.2)	-
Pain	-	-	3 (6.1)	
Pulmonary radiation injury	-	-	1 (2)	1 (2)
Radiation mucositis	-	-	1 (2)	-
Pneumonitis	-	-	1 (2)	-

Acute renal failure observed in 1 patient (G3 at induction).

Acute radiation pneumonitis at 30 days post-completion observed for 1 patient.

## Discussion

Over the last decades, the standard treatment for unresectable locally advanced NSCLC has evolved, from radiotherapy alone [12], to full dose up-front concomitant CT-RT. Two meta-analyses reported that both sequential CT-RT and concomitant CT-RT provide a benefit over radiotherapy alone [13,14]. However, the prognosis for stage III NSCLC remains poor with a 5-year survival rate of less than 20% with CT-RT. The study by Furuse was the first phase III showing a significantly higher median survival in the concomitant arm compared to the sequential arm (16.5 months vs. 13.3 months; p = 0.04) and 2-year survival rates of respectively 34.6% and 27.4%, confirmed by a significantly better 5-year survival (15.8% vs. 8.9%). In this trial using the MVP (mitomycin/vindesine/cisplatin) triplet, myelosuppression was significantly more frequent in the concomitant arm (p < .0001), with surprisingly identical esophageal toxicity [15]. A meta-analysis on 1,205 patients with unresectable locally advanced NSCLC confirmed that, compared to sequential CT-RT, concomitant CT-RT improves 5-year survival from 10.6% to 15.1% (HR = 0.84, [95% CI, 0.74-0.95], p = 0.004). Two-year survival was 30.3% and 35.6% respectively [16]. The question on the potential benefit of induction chemotherapy preceding a concomitant CT-RT remains unanswered.

Subsequent trials mainly used cisplatin plus etoposide with concomitant radiotherapy. However, this doublet was replaced in stage IV patients by more efficient new-generation cisplatin-based doublets (including vinorelbine, taxanes, gemcitabine and more recently pemetrexed).

The phase II CALGB 9431 study first investigated cisplatin/gemcitabine or cisplatin/paclitaxel or cisplatin/intravenous vinorelbine as induction chemotherapy followed by concomitant CT-RT for stage IIIB NSCLC. Median overall survival was 17.7 months for the vinorelbine arm, with 1-year and 2-year survival of 65% and 40% respectively. Grade 3–4 Esophagitis was more frequent with gemcitabine than with vinorelbine [9].

In the NPC 95–01 trial, 3 cycles of cisplatin (120 mg/m^2^ on day 1) plus weekly I.V. 30 mg/m^2^ vinorelbine in the sequential arm, were compared with 2 cycles of cisplatin/etoposide with concomitant radiotherapy, followed by 2 cycles of cisplatin (80 mg/m^2^) plus weekly vinorelbine. Although the difference was not statistically significant due to a lack of power, the median survival of 16.3 months (95% CI, 5.8-34.8) in the concomitant arm was numerically higher than the 14.5 months (95% CI, 8.3-27.4) in the sequential arm (p = 0.24). The 2-year survival rates were 39.3% (95% CI, 29.7-48.9) and 26.5% (95% CI, 17.9-35) respectively. Grade 4 neutropenia was higher in the sequential arm (72% vs. 48%, p = 0.008) as was grade 3–4 esophageal toxicity in the concomitant arm (32% vs. 3%, p < 0.001). Grade 3–4 radiation pneumonitis (11% vs. 5%) was numerically higher in the sequential arm. Toxic deaths in the sequential and concomitant arm were 5.6% and 9.6% respectively [17].

Induction chemotherapy with cisplatin and I.V. vinorelbine followed by concomitant docetaxel and radiotherapy in responding patients had been also assessed in a French phase II trial, the median survival was 13 months [0.3-44.9] [18].

More recently, Horinouchi et al. reported the pool long term results of two consecutive Japanese phase I and II trials, using up-front concomitant chemoradiotherapy with 4 or 3 cycles of I.V. vinorelbine (Day 1 and 8) plus cisplatin. In addition, the second trial also included three cycles of docetaxel consolidation monotherapy. A very high 82.0% objective response rate was reported, whereas the 21.0% 3-year progression-free rate, as the median PFS (13.4 months) were more classical. The pool median overall survival was surprisingly long (30.0 months), but could be explained by inclusion of a large majority of adenocarcinoma patients (64%), as usual in Asian trials, contrasting with the recruitment of our current trial, which included only 35% of adenocarcinoma [19].

In our trial, we chose vinorelbine for its availability in an oral formulation, thus allowing fractionated use without increasing hospital visits, with, as a consequence, a cost-effective impact.

An international phase II trial used induction chemotherapy with oral vinorelbine/cisplatin followed by concomitant CT-RT and oral vinorelbine/cisplatin, with a different schedule, and led to a median survival of 23.4 months (95% CI, 17.6-29.8) and a promising 2-year survival of 48.1%. The toxicity profile showed 27.8% of grade 3–4 neutropenia, 9.3% of grade 3–4 vomiting during the induction chemotherapy, and only 4.3% of grade 3 esophageal toxicity. Two deaths occurred after 2 cycles (massive hemoptysis, cardiac failure) [20].

More recently, the French GFPC 05–03 phase II study assessed induction chemotherapy but with docetaxel/cisplatin, followed by concomitant oral vinorelbine/cisplatin and radiotherapy. Median survival was 20.8 months (95% CI, 13.7-24.1) while 1-year and 2-year survivals were 66.1% (95% CI, 52.1-76.8) and 37.1% (95% CI, 23.3-50.9) respectively. During the induction chemotherapy, grade 3–4 neutropenia was 28.6%, 6 patients stopped treatment for toxicity and 1 patient died. During the CT-RT, 3 patients had grade 3 esophagitis and only one patient had graded 2 radiation pneumonitis (2.6%) [21].

Conversely, we chose to avoid strategies based on adding a consolidation or a maintenance therapy since two phase III trials had been terminated early on the evidence of futility of treatments and an increase of toxicities. One trial evaluated consolidation docetaxel after concomitant CT-RT and the other evaluated maintenance therapy by gefitinib after consolidation docetaxel following concomitant CT-RT [22,23].

In our study, we evaluated induction chemotherapy with vinorelbine/cisplatin followed by concomitant CT-RT in unresectable stage III NSCLC with the same regimen. We used intravenous and oral vinorelbine during the IP and only oral vinorelbine for the CT-RT. The objective ORR of 50.0%, as determined by an independent radiologist, was comparable to the ORR of 41.1% in Descourt et al. trial and 53.7% [95% CI, 39.6-67.4] in Krzakowski et al. study [19,20]. Also, the DCR of 81.4% was high. The ORR was consistent with the median survival of 17.08 months, and the 1-year and 2-year survivals of 68.6% and 37% respectively. These efficacy results are similar to those reported in recent trials and also in other studies which evaluated vinorelbine/cisplatin using the same therapeutic strategy.

As mentioned for the Japanese trial, caution must be applied as the proportion of patients regarding tumor histology differed in the various published trials and this may have confounded the comparison.

Whereas concomitant CT-RT is known to increase radiation toxicities, in our trial the treatment was well tolerated and no grade 3 or 4 esophagitis was reported, suggesting a better esophageal toxicity profile for vinorelbine, as previously reported for vindesine in MVP regimen. We only observed 8.6% grade 3–4 leukopenia, 24.3% grade 3–4 neutropenia, and 2.85% grade 4 febrile neutropenia. Treatment discontinuation due to toxicity was reported in 5 patients, at the end of the IP, but none during the CP. Only one patient had a grade 3 radiation pneumonitis. This favorable toxicity profile is important for quality of life and is not associated with a lower efficacy as may have been feared.

Therefore, this regimen including oral vinorelbine seems to offer a favorable safety profile coupled with similar efficacy when used concomitantly with RT.

## Conclusion

In conclusion, this new fractionated fixed dose administration of oral vinorelbine concomitantly with cisplatin and radiotherapy, following induction vinorelbine/cisplatin is feasible, and offers a well-tolerated (in terms of esophageal and hematological toxicities) therapeutic option, in non-operable IIIA-IIIB NSCLC. The activity of this oral regimen seems comparable to most data previously published with I.V. vinorelbine regimens. However, a trial comparing concomitant oral vinorelbine/cisplatin and radiotherapy, preceded or not by induction vinorelbine/cisplatin, with concurrent I.V. vinorelbine/cisplatin and radiotherapy would be now needed, to confirm these encouraging results and to assess cost-effectiveness of such an out-patient regimen.

## Competing interest

Dr Vincent Le Pennec has received honoraria from FABRE. Dr Christine Dujon has received a grant from FABRE. Bénédicte Precheur Agulhon is employee in the “Institut de Recherche Pierre Fabre”. For the remaining authors none were declared.

## Authors’ contributions

Conception/Design: EL. CT-scan review: VLP Data interpretation: DL, EL, GZ. Manuscript writing: DL, GZ. Final approval of the manuscript: DL, AR, ED, CC, CD, RS, AL, VLP, EF, JC, FM, SD, P, EL, GZ. All authors read and approved the final manuscript.

## Pre-publication history

The pre-publication history for this paper can be accessed here:

http://www.biomedcentral.com/1471-2407/14/231/prepub

## Supplementary Material

Additional file 1 Supplemental Digital Content 1 Drug exposure (ITT population, n = 70). Click here for file

Additional file 2 Supplemental Digital Content 2 Sites of progression (ITT population, n = 70). Click here for file

Additional file 3 Supplemental Digital Content 3 Overall survival rates according to histological type and disease stage (ITT population, n = 70). Click here for file
