# Supplementary material for: A phase II study of cisplatin with intravenous and oral vinorelbine as induction chemotherapy followed by concomitant chemoradiotherapy with oral vinorelbine and cisplatin for locally advanced non-small cell lung cancer
Source: BMC Cancer. 2014 Mar 30;14:231. doi: 10.1186/1471-2407-14-231 (PMC3986598; doi:10.1186/1471-2407-14-231)
Supplement: Additional file 1 — Supplemental Digital Content 1 Drug exposure (ITT population, n = 70). [file 1471-2407-14-231-S1.doc]

**Additional file 1 Supplemental Digital Content 1. Drug exposure (ITT population, n=70)**

|  | **Induction treatment** | **Consolidation treatment** |
| --- | --- | --- |
| Number of treated patients | 70 | 49 |
| Total number of cycles (237) | 139 | 98 |
| Duration of treatment (weeks)  Median | 6.4 [3.0-8.7] | 6 [0.0-7.0] |
| Oral vinorelbine  Median dose intensity, mg/m^2^/week  Median relative dose intensity (%) | 18.2 [0-22.5]  90.8 [0.0-112.7] | 20 [14.3-24.4]  100 [71.4-122.1] |
| IV vinorelbine  Median dose intensity, mg/m²/week  Median relative dose intensity | 7.8 [5.7-9.2]  94.2% [68.2-110.9] | -  - |
| Cisplatin  Median dose intensity mg/m²/week  Median relative dose intensity of Cisplatin | 24.8 [16.1-28.0]  93% [60.4-104.9] | 26.6 [13.3-27.6]  99.6 [49.7-103.2] |
| Radiotherapy  Median cumulative dose, Gy |  | 66 [50-67] |
