# Supplementary material for: A phase II study of cisplatin with intravenous and oral vinorelbine as induction chemotherapy followed by concomitant chemoradiotherapy with oral vinorelbine and cisplatin for locally advanced non-small cell lung cancer
Source: BMC Cancer. 2014 Mar 30;14:231. doi: 10.1186/1471-2407-14-231 (PMC3986598; doi:10.1186/1471-2407-14-231)
Supplement: Additional file 2 — Supplemental Digital Content 2 Sites of progression (ITT population, n = 70). [file 1471-2407-14-231-S2.doc]

**Additional file 2** Supplemental Digital Content 2. Sites of progression (ITT population, n=70)

| **Site** | **N (%)** |
| --- | --- |
| Local progression only | 16 (22.9) |
| Brain | 12 (17.1) |
| Liver | 6 (8.6) |
| Bone | 6 (8.6) |
| Abdominal | 3 (4.3) |
| Renal | 2 (2.3) |
| Nose Ear Throat | 1 (1.4) |
| Clinical progression (no site identified) | 9 (12.9) |
