# Supplementary material for: A phase II study of cisplatin with intravenous and oral vinorelbine as induction chemotherapy followed by concomitant chemoradiotherapy with oral vinorelbine and cisplatin for locally advanced non-small cell lung cancer
Source: BMC Cancer. 2014 Mar 30;14:231. doi: 10.1186/1471-2407-14-231 (PMC3986598; doi:10.1186/1471-2407-14-231)
Supplement: Additional file 3 — Supplemental Digital Content 3 Overall survival rates according to histological type and disease stage (ITT population, n = 70). [file 1471-2407-14-231-S3.doc]

**Additional file 3** Supplemental Digital Content 3. Overall survival rates according to histological type and disease stage (ITT population, n=70)

| **Characteristics** | **Survival at 12 months** | **Survival at 18 months** |
| --- | --- | --- |
| Squamous NSCLC, % | 48.4 [95%CI, 30.2-64.4] | 34.2 [95%CI, 18.0-51.1] |
| Non squamous NSCLC | 30.8 [95%CI, 17.3-45.4] | 28.2 [95%CI, 15.3-42.7] |
| Stage IIIA | 55 [95%CI, 31.3-73.5] | 40 [95%CI, 19.3-60.0] |
| Stage IIIB | 33.3 [95%CI, 20.6-46.6] | 28.8 [95%CI, 16.8-42.0] |
